# ^1^H and ^31^P MRS Interleaved With High Time Resolution Reveals Closely Matching Creatine CH₂ and PCr Dynamics During Exercise

**DOI:** 10.1002/nbm.70132

**Published:** 2025-09-03

**Authors:** Radka Klepochová, Fabian Niess, Matthäus Metz, Barbara Ukropcová, Jozef Ukropec, Siegfried Trattnig, Alexandra Kautzky‐Willer, Martin Krššák, Martin Meyerspeer

**Affiliations:** ^1^ Department of Internal Medicine III, Division of Endocrinology and Metabolism Medical University of Vienna Vienna Austria; ^2^ High‐Field MR Center, Department of Biomedical Imaging and Image‐Guided Therapy Medical University of Vienna Vienna Austria; ^3^ Institute of Experimental Endocrinology, Biomedical Research Center Slovak Academy of Sciences Bratislava Slovakia; ^4^ Institute of Measurement Science Slovak Academy of Sciences Bratislava Slovakia; ^5^ Faculty of Medicine, Institute of Pathophysiology Comenius University Bratislava Slovakia; ^6^ High‐Field MR Center, Center for Medical Physics and Biomedical Engineering Medical University of Vienna Vienna Austria

**Keywords:** creatine visibility, human volunteers, PCr and Cr–CH_2_ exercise‐on kinetics rate, PCr and Cr–CH_2_ recovery rate, proton and phosphorus magnetic resonance spectroscopy, skeletal muscle metabolism

## Abstract

The rate of intramuscular phosphocreatine (PCr) depletion and recovery in response to exercise estimated from ^31^P MRS is an established measure for oxidative capacity. The creatine CH_2_ resonance in ^1^H MRS is known to exhibit a similar pattern. So far, repeating the exercise for consecutive ^1^H and ^31^P experiments posed limitations on the interpretation. Acquiring both datasets in a single time‐resolved experiment allows for direct quantitative comparison of creatine‐CH_2_ and PCr kinetics. This can help answer to what extent creatine‐CH_2_ mimics PCr dynamics and provide data on the visibility of myocellular creatine. Twenty‐seven volunteers, assigned to a group with lower BMI (*n* = 16, BMI = 22.1 ± 3.2 kg/m^2^, age = 35.7 ± 9.3 years) or higher BMI (*n* = 11, BMI = 34.1 ± 3.6 kg/m^2^, age = 35.0 ± 6.6 years) were measured on a 7 T MR system and MR‐compatible ergometer. Localized ^1^H and ^31^P MR spectra were acquired interleaved during a single 5‐min submaximal exercise effort and recovery, with 6 s time resolution. Exercise led to reduced creatine‐CH_2_ signal, while the CH_3_ resonance remained stable. Neither the recovery nor exercise‐on‐kinetics time constants were significantly different when quantified from ^1^H or ^31^P MR spectra in each group (recovery, lower BMI: τ_PCr‐recovery_ = 35 ± 12 s vs. τ_Cr–CH2‐recovery_ = 36 ± 11 s, higher BMI: τ_PCrrecovery_ = 65 ± 30s vs. τ_Cr–CH2‐recovery_ = 60 ± 11 s, and exercise‐on, lower BMI: τ_PCr‐on‐kinetics_ = 39 ± 14 s vs. τ_Cr–CH2‐on‐kinetics_ = 38 ± 15 s, higher BMI: τ_PCr‐on‐kinetics_ = 77 ± 53 s vs. τ_Cr–CH2‐on‐kinetics_ = 70 ± 53 s). Significantly different time constants between the groups distinguished by BMI were detected, likewise with ^1^H and ^31^P MRS. Interestingly, though, creatine‐CH_2_ and PCr depletion differed, correlating positively. Closely matching Cr–CH₂ and PCr kinetics was confirmed for the first time in single time‐resolved experiments, using interleaved ^1^H and ^31^P MRS. The strong correlation between τ_PCr_ and τ_Cr–CH₂_ and preserved intergroup differences suggests that quantifying Cr–CH₂ by ^1^H MRS might, within limitations, serve as a surrogate for the estimation of oxidative capacity via PCr from ^31^P MR spectra. The results contribute to the discussion on NMR visibility of myocellular creatine pools.

## Graphical abstract

The kinetics of the creatine CH_2_ resonance (Cr‐CH_2_) and phosphocreatine (PCr) during muscle exercise and recovery were studied using interleaved ^1^H and ^31^P MRS at 7T in 27 volunteers. The two methods exhibit no differences in depletion and recovery time constants. A comparison between participants with lower and higher BMI showed different kinetics with both methods. The strongly correlating recovery time constants suggest that ^1^H MRS could, within limits, serve as a surrogate for oxidative capacity estimated via PCr dynamics.

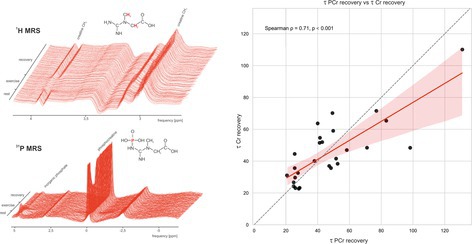

**Abbreviations**
^1^H MRS: proton magnetic resonance spectroscopy
^31^P MRS: phosphorus magnetic resonance spectroscopy
ATP: adenosine triphosphate
Cr: creatine
Cr–CH_2_
: creatine methylene
Cr–CH_3_
: reatine methyl
PCr: phosphocreatine
Pi: inorganic phosphate
Q_max_
: mitochondrial capacity
SNR: signal‐to‐noise
VOI: volume of interest
τ_PCr on‐kinetics_
: PCr depletion rate during exercise
τ_PCr recovery_
: PCr resynthesis rate during recovery

## 1 Introduction

Localized proton ^1^H and phosphorus ^31^P magnetic resonance (MR) spectra of human muscle are strongly affected by exercise. Adenosine triphosphate (ATP) supply and demand are buffered by phosphocreatine (PCr) via the creatine‐kinase reaction [1], both ^31^P MRS metabolites are prominently visible in spectra. This is the basis for ^31^P MRS having become a well‐established method to investigate skeletal muscle energy metabolism in vivo [2]. PCr levels measured by ^31^P MRS decrease during exercise and recover towards basal values after exercise. PCr recovery is almost completely fuelled by oxidative ATP synthesis; hence, the rate at which PCr is replenished can serve as a surrogate of oxidative capacity. A faster recovery of PCr post‐exercise signifies swifter replenishment and, consequently, superior mitochondrial function in restoring cellular energy. However, for using ^31^P MRS on an MR scanner, it has to be equipped with multi‐nuclear MR capabilities in hard‐ and software, which is not the case for most routine clinical installations.

In contrast, ^1^H MRS is readily available with clinical MRI systems or can be enabled as a software option. The ^1^H MR spectrum of skeletal muscle exhibits two creatine (Cr) signals, the methyl (Cr–CH_3_) resonance at 3.02 ppm and the methylene (Cr–CH_2_) peak at 3.96 ppm. Interestingly, exercise‐induced PCr depletion also has a strong influence on the spectral appearance of the Cr–CH_2_^1^H MRS resonance [3]. This was first reported by Kreis et al. already in 1999, who showed that intensive exercise resulting in near exhaustion leads to a strong reduction to virtual disappearance of the Cr–CH_2_ resonance in ^1^H MR spectra, while the Cr–CH_3_ peak was largely unaffected by exercise [3]. This observation was interpreted as the Cr–CH_2_ resonance representing the visible fraction of phosphorylated PCr content in ^1^H MRS spectra; however, the exact mechanism explaining this effect has not been fully understood [3, 4].

Detection of Cr signals using ^1^H single‐voxel MRS and the exercise‐challenged time course of PCr and Pi concentrations using dynamic ^31^P MRS are well established and there is good consensus about their standardization [5, 6]. Acquiring ^1^H and ^31^P MRS information in two separate experiments, and hence repeating the exercise for each nucleus, presents certain limitations regarding the reproducibility of physiologic conditions. Thus, this study aims to investigate whether we can simultaneously observe the ^31^P and ^1^H time courses within a single experiment. By performing both measurements in one session, we aim to avoid potential confounding effects associated with repeated exercise bouts, such as fatigue, incomplete pH recovery, and altered perfusion, which may influence the parameters of subsequent measurements. Additionally, repeating exercise trials exactly the same is challenging, difficult to quantify, and, in the strictest sense, not feasible. To achieve this, we employ interleaved ^31^P/^1^H MRS [7, 8, 9, 10] ensuring high signal‐to‐noise (SNR) ratios for both spectra by utilizing a 7 T MR scanner and a radiofrequency coil [11] optimized for human calf imaging. This brings the potential to reveal direct correlations between Cr (as measured by ^1^H MRS) and PCr (by ^31^P MRS) response to the exercise. If Cr–CH_2_ depletion reflects oxidative metabolism, ^1^H MRS would in principle have the potential to render non‐invasive metabolic studies more widely accessible, as it does not require multi‐nuclear MRI capabilities.

In this work, we used 7 T MRS with a dedicated RF coil for its enhanced precision and time resolution, with co‐localization, and utilized the system's capability of interleaving ^31^P and ^1^H acquisitions to directly compare time courses of PCr and Cr–CH_2_ acquired simultaneously during exercise and recovery in human subjects. The primary aim was to investigate skeletal muscle oxidative metabolite kinetics through time‐resolved ^1^H MRS. Additionally, we performed a bivariate analysis of the interrelations between Cr–CH_2_ and PCr, derived from ^1^H MRS and ^31^P MRS parameters, respectively. To highlight the value of these interrelations, we included volunteers with obesity, who, as observed previously [12, 13], tend to exhibit slower PCr recovery post‐exercise.

## 2 Material and Methods

### 2.1 Study Population

Thirty‐two volunteers participated in the study. Data quality was sufficient for analysis in 27 volunteers, of which 16 were assigned to a group with a lower body‐mass index (BMI) of 22.1 ± 3.2 kg/m^2^, corresponding to the normal range (age: 35.7 ± 9.3 years, sex: 9 f/7 m) and 11 volunteers to a group with a higher BMI of 34.1 ± 3.6 kg/m^2^ which corresponds to the range of obesity, with one subject falling into pre‐obesity (age: 35.0 ± 6.6 years, sex: 6 f/5 m, waist circumference = 107 ± 12 cm, VO₂_max_ [from cycle spiroergometry] = 23 ± 6 mL kg^−1^ min^−1^). Individuals with any chronic disease or regular use of pharmacotherapy were not eligible to enter the study. Data from five subjects had to be excluded from the analysis due to either poor shim (water linewidth after shim > 70 Hz, *n* = 3), insufficient water suppression (*n* = 1) or baseline distortion (*n* = 1) in the ^1^H spectra, all presumably caused by dislocation during the exercise. The study was approved by the local Ethics Committee, and it conforms to ethical guidelines of the Declaration of Helsinki, as revised in 2024. All individuals were informed about the study protocol and signed a written informed consent prior to study entry.

### 2.2 Magnetic Resonance Measurements

Measurements were performed in the morning after overnight fasting in a single exercise–recovery session. Volunteers were positioned in a supine position with the right calf placed on the RF coil, which was installed on a non‐magnetic ergometer inside the scanner. In detail, an in‐house‐built dedicated ^1^H (2‐channel)/^31^P (3‐channel) surface coil transceiver array [11] was used in a whole‐body 7 T MR system (Magnetom 7 T Plus, Siemens Healthcare GmBH, Erlangen, Germany). An MR‐compatible ergometer (Trispect, Ergospect, Innsbruck, Austria) was used for in‐magnet plantar flexion exercise.

**FIGURE 1:**
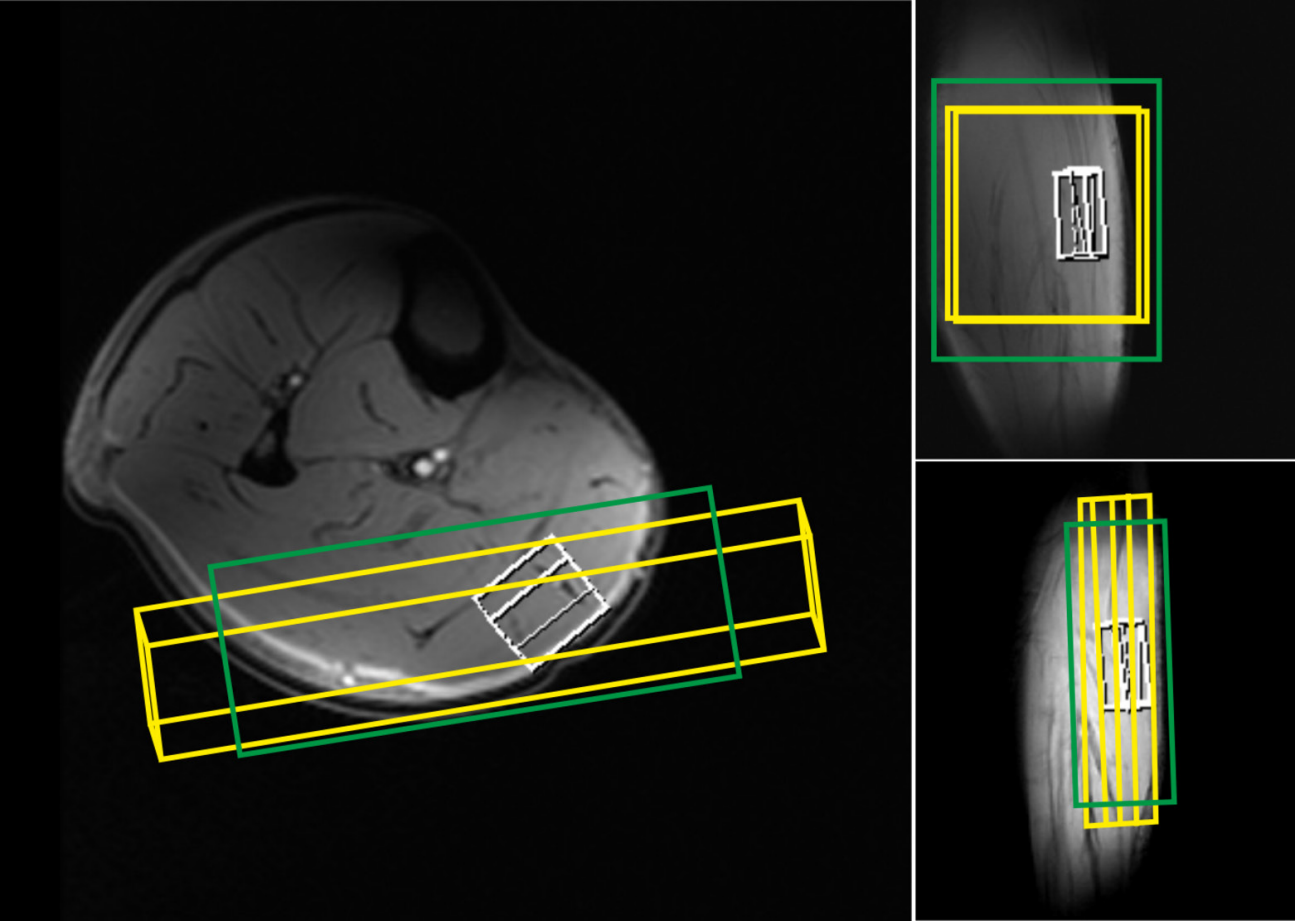
Localizer image of a human calf indicating the localization of VOIs. Yellow: slice selected for ^31^P MRS using DRESS (a slab only localized in A‐P‐oblique direction), white: 3D‐localized voxel for ^1^H MRS using semi‐LASER, green: shim volume.

The volume of interest (VOI) for ^1^H MRS Cr detection (13 × 20 × 40 mm^3^) and an 18‐mm slab for ^31^P MRS for PCr and inorganic phosphate (Pi) detection was carefully placed predominantly within the gastrocnemius medialis and lateralis muscles (Figure 1). Second‐order shimming was done with the method implemented by the manufacturer supplemented with interactive shim, resulting in a water linewidth of 37 to 43 Hz (magnitude spectra) in the region of interest (Figure 1, green box).

For quantification purposes, a separate unsuppressed water spectrum was acquired in a voxel with the same size as used for Cr–CH_2_ measurements. The linewidth of the water peak in the real spectrum was 20.0 ± 4.9 Hz, and the creatine CH_2_ peak linewidth was 5.5 ± 3.5 Hz.

Dynamic localized MRS data were acquired using 3D‐localized ^1^H semi‐LASER [14, 15] (TE = 30 ms) and slab‐localized ^31^P DRESS [16]. The sequences were played out in an interleaved fashion with a TR of 6 s in one exercise/recovery session (3 min rest, 5 min exercise at 30% of maximal voluntary contraction force, 10 min recovery), similarly as published [9]. Other sequence parameters were (equally for ^1^H and ^31^P MRS): excitation flip angle = 90°, receiver bandwidth = 2500 Hz, acquisition duration = 819 ms. The volunteers were pushing the pedal twice per TR, audio‐cued by gradient noise, to ensure that data were acquired when the muscle was relaxed between pedal pushes.

To determine maximum voluntary force, the participants were asked to perform 4 to 6 maximal isometric plantarflexion pushes against a solid block in the same position as used inside the MR scanner. During these trials, the force at the ball of the foot was measured using a dynamometer. The pedal's recoil force was then set to 30% of this measured maximum force by adjusting the pressure in the ergometer system. This pressure setting was then maintained automatically throughout the exercise protocol, ensuring consistent resistance and adherence to the prescribed intensity.

### 2.3 Data Processing

^1^H/^31^P spectroscopy data were processed from raw data using in‐house‐developed Python scripts (http://www.python.org). This involved phasing the signal of each channel by referencing the highest peak and weighting each channel by this peak's amplitude before calculating the sum over all channels. The reference peaks used were (resting) PCr for ^31^P MRS, the water peak in the unsuppressed water spectrum, and the EMCL CH_2_ peak in the water‐suppressed ^1^H spectrum. Peak amplitudes were quantified with AMARES, using jMRUI v6.0 alpha. For fitting the 3.9 ppm Cr–CH_2_ resonance, HLSVD peak removal over the lipid resonances at 1.5 ppm was used (HLSVD no‐max‐2048‐points filter, 5 components).

For absolute quantification of the visible fraction of the Cr–CH₂ resonance, the fully‐relaxed water signal was measured separately. Concentrations were calculated in mmol/L (tissue volume) units, by

$$C_{m}=C_{H_{2}O}\times \left(\frac{S_{m}}{S_{H_{2}O}}\right)\times \left(\frac{n_{H_{2}O}}{n_{m}}\right)\times \left(\frac{R_{H_{2}O}}{R_{m}}\right)\times W_{H_{2}O}\times \rho_{\text{muscle}}$$

with *S* the signal intensity of water or Cr, *n*_H2O_ and *n*_m_ the number of equivalent protons (= 2), *R* the correction factors for *T*_1_ and *T*_2_ relaxation (H_2_O: *T*_1_ = 3334 ms, *T*_2_ = 27.1 ms, Cr‐CH₃: *T*_1_ = 1496 ms, *T*_2_ = 166 ms) based on relaxation times measured in the vastus lateralis muscle, which closely resembles the gastrocnemius in muscle fiber composition and function [17], *C*_H2O_ = 55.56 mol/L the water concentration, *W*_H2O_ the approximate water content in skeletal muscle tissue (0.77 L/kg_ww_), and *ρ*_muscle_ the specific weight of skeletal muscle tissue (1.06 kg/L).

^31^P MRS yielded PCr and Pi concentrations (quantification of ^31^P metabolites was performed by normalizing to the γ‐ATP peak, assuming a constant concentration of 8.2 mmol/L in skeletal muscle, quantified from 20 spectra averaged at rest, et the end of exercise and at the end of recovery), relative PCr depletion and depletion rate during exercise (*τ*_PCr on‐kinetics_) and resynthesis rate during recovery (*τ*_PCr recovery_), the maximal oxidative phosphorylation rate, that is, mitochondrial capacity (*Q*_max_), and the time course of intracellular pH [18, 19]. τ_Cr–CH2 on‐kinetics_ and τ_Cr–CH2 recovery_, as well as the corresponding time constants based on [PCr], were fitted with a mono‐exponential function using curve fitting in MATLAB. A *T*₁ correction was applied based on literature‐reported *T*₁ values for PCr, Pi, ATP, PDE, and others, using a repetition time (TR) of 6 s [6].

For the calculation of PCr and Cr–CH_2_ depletion (%), values were taken as the average during the last 20 measurements (2 min) at the end of exercise compared to the end of recovery value, which was averaged over the final 20 measurements (2 min).

*Q*_max_ was calculated using the Michaelis–Menten model based on ADP concentration, using the equation

$$Q_{\max}=V_{\mathrm{PCr}}\left(1+\frac{K_{m}}{\left[\mathrm{ADP}\right]}\right)$$

where *V*_PCr_ is the initial rate of PCr recovery, *K*_m_ is the Michaelis constant (assumed to be 0.03 mM), and [ADP] is the steady‐state ADP concentration in μM.

To assess the robustness of signal quantification, we compared the SNR in ^1^H MRS and ^31^P MRS. SNR was calculated in the frequency domain as the amplitude of the Cr–CH₂ peak in ^1^H MRS and the PCr peak in ^31^P MRS, divided by the root mean square (RMS) of noise estimated from an artifact‐free region of the spectrum. No line broadening or zero‐filling was applied prior to SNR estimation.

Group and method comparisons for each parameter were performed using Mann–Whitney *U* tests. All values are provided as mean ± standard deviations, and, in Table 1, range, median, interquartile range (IQR) and *p* values from Mann–Whitney *U* tests are given additionally.

**TABLE 1:** Results from ^1^H and ^31^P MRS. Mean, standard deviation (stdev, range, min, quartile 1 [Q1], median, quartile 3 [Q3], max and interquartile range [IQR]) are provided.

		^31^P MRS PCr rest /mM	^1^H MRS Cr2 rest /mM	^31^P MRS PCr ex /mM	^1^H MRS Cr2 ex /mM	^31^P MRS PCr rec /mM	^1^H MRS Cr2 rec/ mM	^31^P MRS PCr depletion /%	^1^H MRS Cr2 depletion /%	^31^P MRS τ_rec_/s	^1^H MRS τ_rec_ /s	^31^P MRS τ_on‐kinetics_ /s	^1^H MRS τ_on‐kinetics_ /s	pH_ex_	pH_rest_	Q_max_ /mM s^−1^
Group with lower BMI (*n* = 16)	**mean**	**34.6**	**7.7**	**22.8**	**4.6**	**33.6**	**8.2**	**31.8**	**47.9**	**35.2**	**35.7**	**38.8**	**38.0**	**7.01**	**7.03**	**0.57**
	**stdev**	**4.6**	**6.5**	**4.6**	**5.1**	**4.6**	**7.5**	**12.9**	**22.9**	**12.3**	**11.0**	**14.1**	**14.8**	**0.03**	**0.01**	**0.22**
	**range**	14.5	22.6	15.4	19.1	16.6	24.8	35.2	80.3	37.6	36.4	46.0	52.8	0.13	0.06	0.79
	**min**	27.9	1.1	15.3	0.1	25.8	1.4	20.2	18.3	20.6	22.6	22.0	17.7	6.96	7.01	0.33
	**Q1**	31.2	2.5	19.5	1.1	31.5	2.8	22.4	31.8	25.5	26.0	27.2	27.1	7.00	7.02	0.39
	**median**	**33.9**	**6.6**	**23.9**	**3.2**	**33.0**	**6.2**	**26.5**	**44.1**	**28.3**	**35.6**	**38.2**	**36.5**	**7.01**	**7.03**	**0.54**
	**Q3**	36.2	11.0	25.6	6.2	35.7	11.7	39.3	54.5	47.5	42.3	47.4	47.7	7.03	7.04	0.64
	**max**	42.4	23.7	30.6	19.1	42.4	26.2	55.4	98.5	58.3	59.0	68.0	70.5	7.09	7.07	1.11
	**IQR**	5.0	8.5	6.0	5.1	4.3	8.9	16.9	22.7	20.0	16.3	20.2	20.5	0.03	0.02	0.25
Group with higher BMI (*n* = 11)	**mean**	**31.2**	**5.0**	**15.4**	**2.1**	**29.1**	**5.3**	**47.3**	**63.9**	**64.9**	**59.5**	**77.1**	**70.0**	**7.00**	**7.05**	**0.36**
	**stdev**	**4.7**	**3.9**	**6.1**	**3.0**	**6.9**	**4.1**	**17.5**	**20.3**	**30.7**	**20.9**	**53.7**	**52.9**	**0.07**	**0.03**	**0.21**
	**range**	12.5	13.2	23.8	10.5	22.9	14.1	49.2	69.0	103.5	77.6	194.5	170.7	0.24	0.11	0.75
	**min**	26.3	0.6	4.6	0.2	16.0	0.5	22.9	26.6	27.5	32.5	31.1	19.5	6.85	6.97	0.10
	**Q1**	27.2	2.0	12.4	0.5	25.8	1.9	33.9	53.0	42.1	48.4	47.8	32.2	6.97	7.04	0.26
	**median**	**30.0**	**5.0**	**15.3**	**1.0**	**30.1**	**5.7**	**47.0**	**58.6**	**52.5**	**54.6**	**57.9**	**44.4**	**7.02**	**7.05**	**0.32**
	**Q3**	34.5	6.7	18.2	2.4	33.6	7.0	60.5	81.4	80.0	67.7	84.4	86.7	7.05	7.06	0.37
	**max**	38.8	13.8	28.47	10.7	38.8	14.6	72.1	95.6	131.0	110.1	225.5	190.2	7.08	7.08	0.85
	**IQR**	7.4	4.7	5.8	1.8	7.7	5.1	26.6	28.4	37.9	19.4	36.7	54.6	0.08	0.02	0.11
*U* test btw. groups (*p* value)		0.720	0.267	0.002	0.121	0.072	0.389	0.020	0.052	0.004	0.001	0.004	0.144	1.00	1.00	0.006
*U* test btw. methods (*p* value)											0.944		0.417			

*Note:* Mann–Whitney *U* tests' *p*‐values are included.

Abbreviations: Cr2, Cr‐CH_2_; ex, end of exercise; rec, recovery.

To assess the relationship between the two measurement modalities (^1^H MRS and ^31^P MRS), correlation analyses were conducted using Spearman's correlation coefficient, selected according to data normality. All statistical tests were two‐sided, and significance was set at *p* < 0.05.

## 3 Results

**FIGURE 2:**
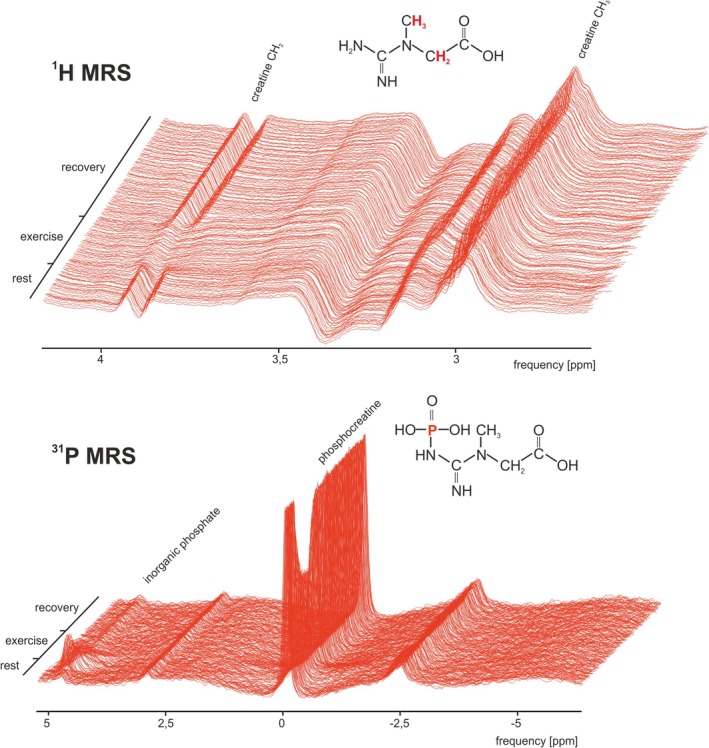
Representative time course of ^1^H (top) and ^31^P (bottom) spectra acquired during 3 min at rest, 5 min of plantar flexion exercise, and 10 min of recovery from the gastrocnemius muscle of one volunteer. In the structural formulae of (P)Cr the protons and phosphorus giving rise to the MRS signal are highlighted.

**FIGURE 3:**
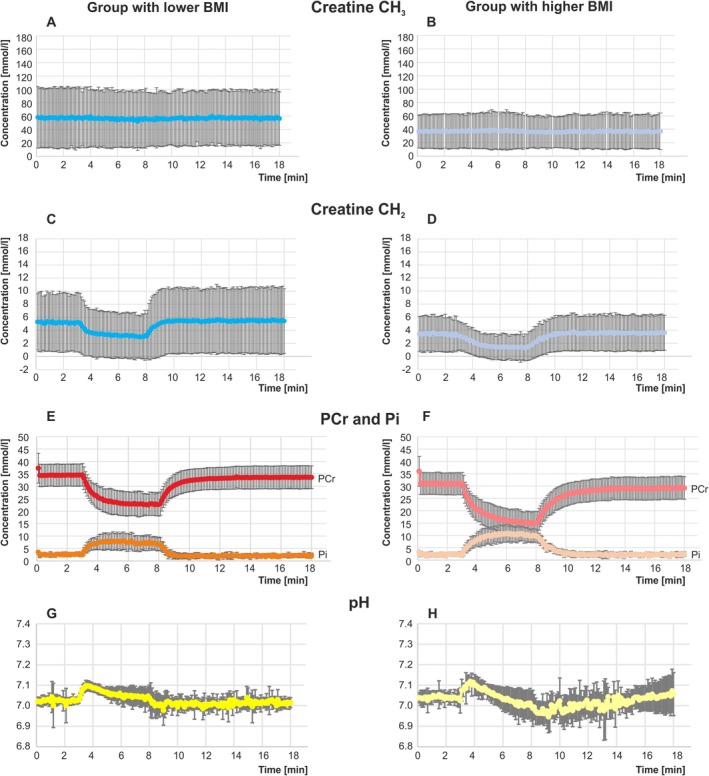
Averaged time series of interleaved measurement of Cr‐CH_2_ [mmol/l] (A, B, C, D), PCr and Pi [mmol/l] (E, F) and pH (G, H) evolution during rest, exercise, and recovery including 16 volunteers from the group with lower BMI (left) and 11 from the group with higher BMI (right).

Submaximal exercise led to a substantial reduction of the Cr–CH₂ resonance at 3.92 ppm in the ^1^H MR spectra (Figure 2 and Figure 3), with only approximately 40% to 50% of the baseline signal remaining, while the Cr–CH₃ resonance at 3.02 ppm remained stable during exercise. This reduction in Cr–CH₂ parallels the exercise‐induced PCr depletion observed in the time‐resolved ^31^P MR spectra, which were acquired interleaved with ^1^H MRS. The SNR of the Cr–CH₂ resonance at rest in ^1^H MRS was 1.05 ± 0.55, whereas the resting PCr SNR in ^31^P MRS was notably higher, at 27.6 ± 6.5 (both quantified without apodization or zero‐filling, in the frequency domain).

With both methods, the recovery time constants τ_Cr–CH2 recovery_ (*p* = 0.001) and τ_PCr recovery_ (*p* = 0.004) were found to be significantly longer in the group with a higher BMI, consistent with reduced *Q*_max_ (*p* = 0.006). Also, the exercise‐on time constant quantified with ^31^P MRS τ_PCr on‐kinetics_ was longer (*p* = 0.004), while differences between τ_Cr–CH2 on‐kinetics_ of the BMI groups did not reach significance (*p* = 0.004).

Comparisons between the two MRS methods (^1^H MRS Cr–CH_2_ and ^31^P MRS PCr) revealed no significant differences in τ_recovery_ (*p* = 0.944) or τ_on‐kinetics_ (*p* = 0.417).

**FIGURE 4:**
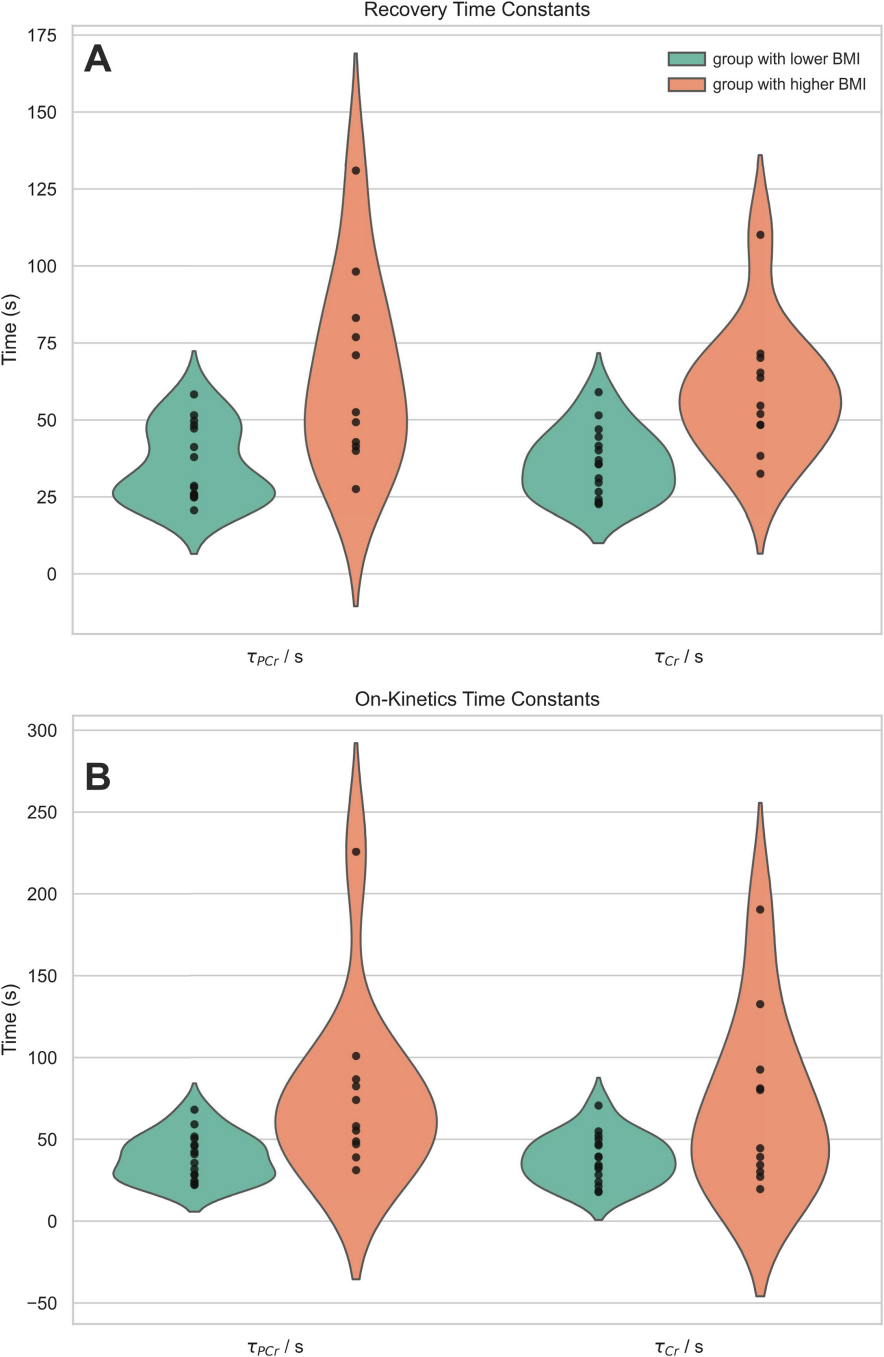
Violin plots of time constants (*τ*) for phosphocreatine (PCr) and creatine (Cr–CH_2_) during recovery (A) and early exercise (exercise‐on kinetics). Green violins represent the lower BMI group, and orange violins represent the higher BMI group.

Numerical results for metabolic concentrations and time constants of exercise‐induced changes across BMI groups and methods are summarized in Table 1. Violin plots in Figure 4 illustrate the distribution and variability of τ_PCr_ and τ_Cr–CH2_ parameters, highlighting data spread and skewness between groups.

**FIGURE 5:**
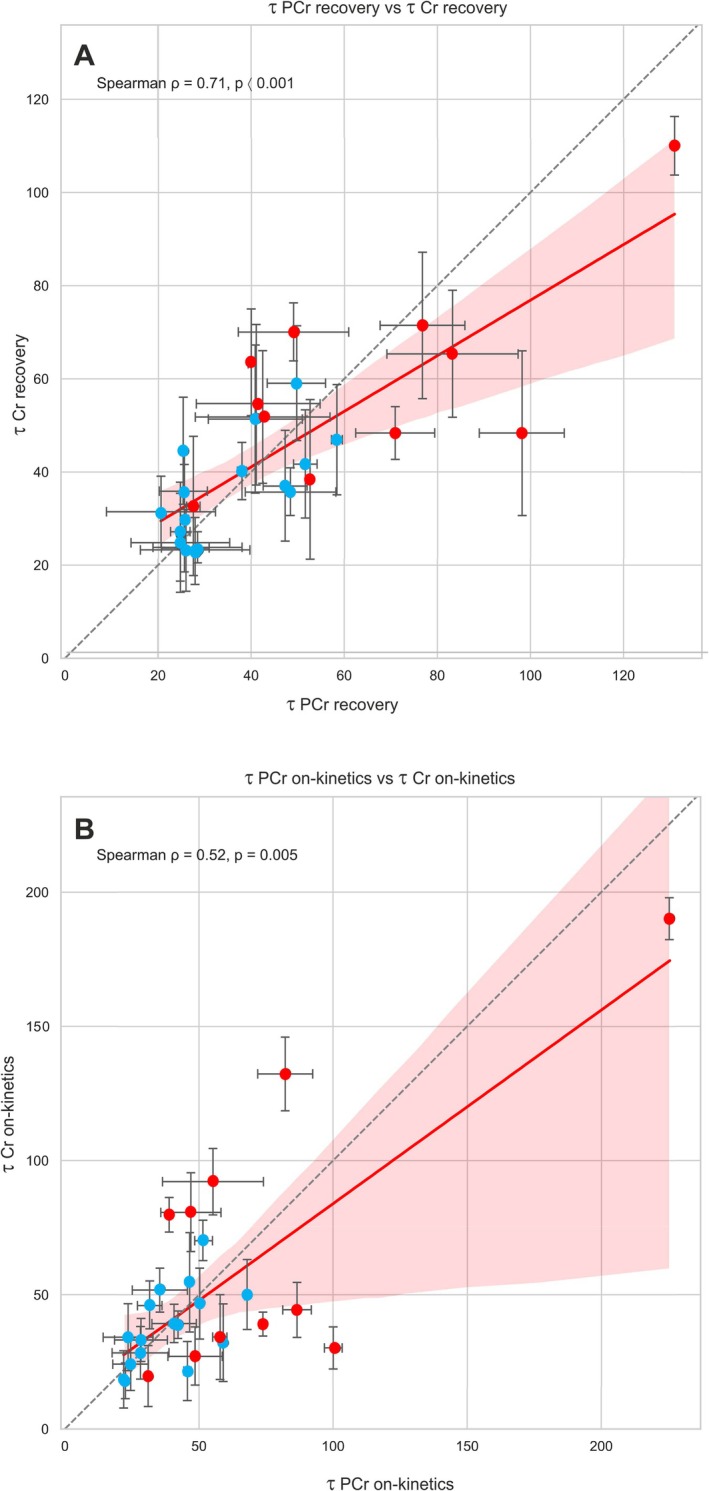
Relationship between individual time constants (τ) of phosphocreatine (τ_PCr_) and creatine (τ_Cr–CH₂_) for recovery (A) and on‐kinetics (B). Each dot represents an individual subject. Blue dots represent participants with lower BMI, and red dots represent those with higher BMI. Horizontal and vertical bars indicate standard squared error (SSE) from individual curve fitting in MATLAB for each τ value. The solid red line illustrates the monotonic trend between τ_Cr–CH₂_ and τ_PCr_, consistent with the Spearman rank correlation (*ρ*), with shaded red areas representing the 95% confidence interval for the fit. The dashed gray identity line serves as a reference to indicate where τ_Cr–CH₂_ would equal τ_PCr_. Significance levels are indicated.

Correlation analysis revealed a strong positive association between *τ*_PCr recovery_ and *τ*_Cr–CH2 recovery_ (Spearman *r* = 0.71, *p* < 0.001) (see Section 4: Discussion and Figure 5). A moderate correlation was also observed between *τ*_PCr on‐kinetics_ and τ_Cr–CH2 on‐kinetics_ (Spearman *r* = 0.52, *p* = 0.005), indicating a fair level of agreement between the two modalities (Figure 5).

## 4 Discussion

This is the first study presenting a per‐subject analysis of time‐resolved skeletal muscle Cr–CH_2_ and PCr quantified during a single exercise effort. This was achieved with interleaved ^1^H/^31^P MRS at 7 T involving in‐magnet plantar flexion exercise. Despite the lower SNR of the Cr–CH_2_ resonance, which made the data analysis challenging, especially during the exercise period, we were able to quantify both time courses in a single experiment in 27 subjects. This allowed for direct comparison of individual Cr–CH_2_ and PCr time courses during exercise and recovery.

Cr–CH₂ and PCr exhibited highly similar kinetic behavior across all participants, with time constants during both early exercise and post‐exercise recovery phases closely matching on group levels.

We included sedentary individuals from a group with an average BMI in the range of obesity because the primary parameter of interest, that is, the kinetics of the apparent Cr–CH_2_ and PCr signal intensity, is expected to show significant differences between this group and controls [12, 18].

The strong positive correlation of recovery time constants (with moderate correlation between exercise‐on time constants) found by Spearman's rank correlation analysis (Figure 5), and that the differences between BMI groups were indeed found significant with both methods, suggests that the time constants quantified from the Cr–CH₂ signal might, within limitations, serve as a surrogate for PCr to investigate impaired oxidative capacity. The quantitative agreement of the time constants found on a group level (Table 1) further suggests that ^1^H MRS‐based estimation of oxidative capacity can yield meaningful data, if SNR permits and as long as pH remains neutral throughout exercise is ensured.

However, it is important to note that Spearman's rank correlation indicates that higher values of τ_Cr–CH₂_ tend to correspond to higher values of τ_PCr_, but does not imply that the absolute values themselves are identical or interchangeable. This distinction is relevant because the absolute value of τ_PCr recovery_ carries quantitative physiological meaning, and τ_Cr–CH_₂ recovery is therefore not a 1:1 replacement but may still serve as a practical proxy in specific settings.

Thus, while ^1^H MRS‐derived τ_Cr–CH₂_ recovery cannot fully replace τ_PCr recovery_ in absolute terms, under certain conditions it may offer a practical alternative or screening tool when direct ^31^P MRS is not feasible.

Despite the strong correlation of exercise‐on and recovery time constants, the magnitude by which Cr–CH₂ and PCr were depleted during exercise differed significantly. This could be attributed to multiple factors: (i) Partial linkage of Cr–CH₂ to PCr: Not all visible Cr–CH₂ may correspond to PCr, with a portion potentially residing in structurally constrained compartments, (ii) Compartmentalization of Cr pools: The structural organization of muscle tissue may result in differential Cr–CH₂ visibility depending on fiber orientation and magnetization transfer effects, (iii) Voxel size and positioning effects: The ^1^H MRS VOI (13 × 20 × 40 mm^3^) was optimized to exclude fat and achieve good water signal suppression, whereas the ^31^P MRS volume was optimized for ^31^P SNR, potentially leading to signal discrepancies.

Ad (i): The observation of diminishing Cr–CH₂ signals during exercise, despite total creatine (the sum of creatine and phosphocreatine) remaining constant, merits discussion. This phenomenon has been explored in previous studies [3, 20, 21], suggesting that structural effects and compartmentalization of metabolite pools contribute to limited visibility of certain signals. Moreover, it has been speculated that the Cr–CH₂ peak may, in part, represent PCr, given that PCr is more abundant than free creatine in human skeletal muscle (approximately 2:1) [22]. Additionally, the Cr–CH₂ peak is relatively small and therefore noisy, making it challenging to accurately assess the creatine contribution using this signal.

Magnetization transfer studies [23, 24, 25] suggest that Cr–CH₂ signal intensity is modulated by exchange processes between PCr and free Cr. This exchange occurs via creatine kinase, which catalyzes the reversible reaction

$$\mathrm{PCr}+\mathrm{ADP}+H^{+}↔\mathrm{Cr}+\mathrm{ATP}.$$

During exercise, PCr depletion leads to an increased free Cr pool, which should theoretically maintain the Cr–CH₂ signal. This means that when PCr is used up, it breaks down into free Cr and phosphate. Since the total creatine (Cr + PCr) should remain constant, one might expect that the Cr–CH₂ signal in ^1^H MRS would stay the same. However, in reality, the Cr–CH₂ signal decreases or disappears, and the effect of exchange between PCr and Cr is very slow and does not significantly affect the shape of the peaks [26].

The observed decrease in Cr–CH₂ signal during exercise suggests that not all creatine is equally detectable by ^1^H MRS. Changes in T₂ relaxation times, local magnetic environment, or molecular mobility could transiently contribute to altered creatine visibility during exercise [5]. Additionally, it is established that ^1^H MR signal sensitivity is influenced by fiber orientation and local field inhomogeneities, both of which may change with contraction [5].

Another attempt to explain the observed effect of exercise on Cr–CH_2_ involves the compartmentalized nature of energy metabolism within muscle cells. During contraction, energy flow is concentrated in certain cellular regions [27] where its signal could be less visible, such as near membranes or within subcellular structures like muscle triads [28]. The reorganization of Cr within muscle fibers during exercise could therefore affect its detectability. During recovery, both PCr and Cr redistribute more evenly throughout the cytoplasm, potentially restoring MR visibility [29, 30].

Ad (ii): The residual dipolar coupling of Cr–CH₂ could further contribute to its selective disappearance via spatial ordering effects and compartmentalization, as its spectral properties are highly orientation‐dependent [21]. The splitting of the creatine doublet is modulated by the orientation of muscle fibers relative to the magnetic field (*B*₀). For instance, a singlet observed in the soleus muscle corresponds to a fiber orientation of approximately 55° relative to B₀, while a splitting of about 10 Hz corresponds to an angle of approximately 35°. This highlights the importance of voxel positioning, but also suggests that changes in visibility—especially during exercise—may stem from interactions at the molecular level, such as those related to residual dipolar coupling or binding to subcellular structures [4].

An animal study [31] provided evidence challenging the hypothesis that the CH₂ resonance arises directly from PCr. In vivo ^1^H MR spectra showed the same ratios between CH_2_ and CH_3_ Cr resonances in wild type and creatine‐kinase deficient animals. In this study, *post‐mortem* ^31^P MRS in wild‐type mice showed complete PCr depletion, while the CH₂ doublet persisted unchanged, suggesting that the CH₂ resonance would reflect total creatine rather than PCr content. This discrepancy may be due to tissue degradation *post‐mortem* affecting molecular exchange rates and dipolar interactions, making these findings less representative for in vivo conditions [31].

This does, however, not contradict any effects that are associated with creatine kinase‐mediated exchange processes in vivo that might well alter the MR visibility of Cr–CH₂. Rather, that this is only observed in vivo supports the idea that it is related to the active role of creatine and creatine kinase in facilitating phosphorus transfer.

Ad (iii): In our study, the same muscles were selected with localization methods, but the volumes contributing to ^1^H and ^31^P signal were different. On the one hand, for quantifying the Cr–CH_2_ resonance in the ^1^H MR spectra, it is important to exclude excessive fat compartments from the volume of interest and to enable sufficient excellent water suppression and shim; hence, a rather small ^1^H single voxel (10.4 cm^3^) was selected. On the other hand, selecting such a small VOI with ^31^P MRS would result in an SNR ratio insufficient for quantification of metabolites with lower concentrations, such as inorganic phosphate. Also, DRESS allows for quantification of ATP, which was used as a concentration reference for absolute quantification of [PCr] and [Pi]. The VOI differences are, however, not likely the primary source of discrepancies in concentration values. Rather, as discussed above, the mechanisms governing MR visibility of creatine (in particular for Cr–CH_2_), which are still not fully clear, are likely the reason for different apparent concentrations. Also, if the volumes selected with ^1^H and ^31^P MRS contain equally exercising tissue (which should be the case here), the relative metabolite depletion should be independent of VOI size. However, different apparent PCr depletion has been reported [32] when grossly different volumes contributed to MR spectra, and to some degree, this might also contribute to the differences in relative metabolite depletion found with the two methods [32].

To further assess the feasibility of ^1^H MRS, a quantitative comparison of SNRs between Cr–CH₂ and PCr signals was performed to better evaluate the feasibility of ^1^H MRS. This analysis confirms that the low SNR of Cr–CH₂ compared to PCr represents a key challenge for using ^1^H MRS to quantify post‐exercise recovery kinetics. Moreover, beyond SNR considerations, the presence of artifacts in the ^1^H spectra constitutes an additional limitation, which requires careful attention.

While ^1^H MRS offers certain practical advantages, such as wider availability on clinical scanners, the relatively low SNR of the creatine CH_2_ resonance may challenge its robustness, especially at lower field strengths and in time‐resolved applications. These findings underscore the need for careful optimization of acquisition protocols if ^1^H MRS is to be used reliably in clinical or field‐strength‐limited environments.

Studies including a higher number of participants will be necessary to account for the higher standard deviation of the on‐kinetics time constant, which arises due to the low ^1^H CH₂ SNR leading to greater uncertainties in fitting, and for verifying assessing skeletal muscle oxidative metabolism by ^1^H MRS.

In this study, neutral end exercise and only mildly acidic post‐exercise pH values were observed via ^31^P MRS. This is crucial because, under these conditions, the simple recovery rate *V*_PCr_ primarily represents oxidative ATP resynthesis. Severe acidification would indicate anaerobic metabolism and could confound the interpretation of PCr resynthesis kinetics. Since ^1^H MRS in the upfield spectral region lacks pH‐sensing capabilities (and the pH‐sensitive resonances in the downfield region of the ^1^H spectrum would require a high number of averages), the application of Cr–CH_2_ time constants is likely best suited for low‐intensity exercise conditions that are expected to remain purely aerobic.

A small but consistent initial increase in intramuscular pH was observed at the onset of exercise. This early alkalinisation is a consequence of PCr splitting via the creatine kinase (CK) reaction as the source of ATP during the initial phase of exercise and has been reported in previous studies using dynamic ^31^P MRS, from our own group and others [2, 6, 33, 34, 35, 36]. The stoichiometry of this reaction results in a net consumption of protons, leading to the observed transient pH increase. As exercise continues, glycolysis and, later, oxidative phosphorylation contribute to ATP production and cause pH to fall. The small dip in the pH curve after exercise results from the reverse process, that is, CK‐mediated PCr production which produces H^+^.

It is also important to consider the potential impact of overnight fasting on muscle metabolism during exercise. Fasting alters substrate availability, typically reducing circulating glucose and glycogen stores while increasing reliance on lipid oxidation. This metabolic shift may lead to reduced glycolytic flux and consequently lower lactate and proton production, potentially influencing the observed pH dynamics. In our study, this may have contributed to the relatively neutral end‐exercise pH values and limited acidification during recovery. While the aerobic nature of the exercise protocol likely played the dominant role, fasting‐induced changes in substrate utilization could further promote oxidative metabolism and attenuate exercise‐induced acidosis. This factor should be taken into account when interpreting our pH and PCr kinetics findings.

Although our group‐level results were highly convincing, individual variability remains a concern. Sensitivity was not sufficient for precise per‐subject quantification of measures of aerobic capacity, suggesting that ^1^H MRS should primarily be used for cohort‐based analyses rather than individual diagnostics.

## 5 Conclusion

Closely matching Cr–CH₂ and PCr kinetics was confirmed for the first time in single time‐resolved experiments, using interleaved ^1^H and ^31^P MRS. The strong correlation between τ_PCr_ and τ_Cr–CH₂_ suggests that Cr–CH₂ could serve as a marker for PCr kinetics, and that, within limitations, ^1^H MRS could be used as a more accessible alternative to ^31^P MRS for estimating oxidative capacity. From the perspective of basic research, the results contribute to the discussion on NMR visibility of creatine pools.

## Author Contributions

**Radka Klepochová:** conception and design of study, acquisition of data, analysis and/or interpretation of data, drafting and revising the manuscript, final approval of the version of the manuscript to be published. **Fabian Niess:** methodological help, data processing script programming, revising the manuscript, final approval of the version of the manuscript to be published. **Matthäus Metz:** methodological help, technical help, revising the manuscript, final approval of the version of the manuscript to be published. **Barbara Ukropcová:** drafting the manuscript, revising the manuscript critically for important intellectual content, final approval of the version of the manuscript to be published. **Jozef Ukropec:** drafting the manuscript, revising the manuscript critically for important intellectual content, final approval of the version of the manuscript to be published. **Siegfried Trattnig:** drafting the manuscript, revising the manuscript critically for important intellectual content, final approval of the version of the manuscript to be published. **Alexandra Kautzky‐Willer:** drafting the manuscript, revising the manuscript critically for important intellectual content, and final approval of the version of the manuscript to be published. **Martin Krššák:** conception and design of study, analysis and/or interpretation of data, drafting and revising the manuscript critically for important intellectual content, final approval of the version of the manuscript to be published. **Martin Meyerspeer:** conception and design of study, analysis and/or interpretation of data, drafting and revising the manuscript critically for important intellectual content, final approval of the version of the manuscript to be published.

## Supporting information


**Data S1:** MRS checklist

## Data Availability

Data available on request from the authors. The data that support the findings of this study are available from the first author upon reasonable request.
