# Supplementary material for: 1H and 31P MRS Interleaved With High Time Resolution Reveals Closely Matching Creatine CH₂ and PCr Dynamics During Exercise
Source: NMR Biomed. 2025 Sep 3;38(10):e70132. doi: 10.1002/nbm.70132 (PMC12406759; doi:10.1002/nbm.70132)
Supplement: Supplementary file 1 — Data S1: MRS checklist [file NBM-38-e70132-s001.doc]

MRS checklist

| 1. Hardware |  |
| --- | --- |
| a. Field strength [T] | *7 T* |
| b. Manufacturer | *Siemens Healthineers, Erlangen, Germany* |
| c. Model (software version if available) | *MAGNETOM 7T Plus | syngo MR E12* |
| d. RF coils: nuclei (transmit/receive), number of channels, type, body part | *Custom-built three channel ^31^P (d = 15 cm, l = 10 cm), two channel ^1^H (d = 17 cm, l = 12.5 cm) transceiver coil, shaped to the human calf, (Ref: Goluch et al. Magn Reson Med 2015;73(6):1190–1195.)* |
| e. Additional hardware | *ergometer from Ergospect dedicated for plantar flexion exercise* |
| 2. Acquisition |  |
| a. Pulse sequence | *Semi-LASER for ^1^H MRS and slab-localized (DRESS) for ^31^P MRS* |
| b. Volume of interest and VOI locations | *^1^H VOI-Single voxel placed obliquely in gastrocnemius medialis and lateralis muscle, avoiding subcutaneous fat, fasciae and adjacent muscles*  *^31^P VOI- slab placed parallel to the coil* |
| c. Nominal VOI size [cm^3^, mm^3^] | *^1^H MRS- Anatomy-matched, 13x20x40 mm^3^*  *^31^P MRS- slab thickness of 18 mm* |
| d. Repetition Time (TR), Echo Time (TE) [ms, s] | *TR = 6 s, TE = 30 ms* |
| e. Total number of excitations or acquisitions per spectrum (NA)  In time series for kinetic studies   1. Number of averaged spectra) per time-point (NA) 2. Averaging method (e.g. block-wise or moving average)   Total number of spectra (acquired / in time-series) | *1 acquisition per spectrum (NA = 1).  Total number of spectra in time series was 180* |
| f. Additional sequence parameters (spectral width in Hz, number of spectral points, frequency offsets)   1. If STEAM:, Mixing Time (TM) 2. If MRSI: 2D or 3D, FOV in all directions, matrix size, acceleration factors, sampling method | *5 kHz, 2048 complex points after removing oversampling* |
| g. Water suppression method | *Method implemented by the manufacturer with a bandwidth of 80 Hz* |
| h. Shimming method, reference peak, and thresholds for “acceptance of shim” chosen | *Second-order shimming was done with the method implemented by the manufacturer supplemented with interactive shim* |
| i. Triggering or motion correction method  (respiratory, peripheral, cardiac triggering, incl. device used and delays) | *Within every TR volunteers were instructed to press the pedal twice between measurements, with noise of the spoiler gradients serving as audio cue, to ensure data acquisition in a relaxed state of the muscle* |
| 3. Data analysis methods and outputs |  |
| a. Analysis software | *All 1H/^31^P spectroscopy data were extracted and processed from raw data using in-house developed Python scripts (http://www.python.org) for phasing and channel combination.*  *Signals were phased to the highest peak magnitude of PCr/ water in the frequency domain after 7 Hz Lorentzian apodization and 4 × zero-filling. The channel combination was then performed by weighted averaging of the raw data (that is, without apodization and zero-filling).*  *All spectra were analyzed with the fitting routine AMARES, using jMRUI v6.0 alpha* |
| b. Processing steps deviating from quoted reference or product analysis software (vendor, version) | *HLSVD no-max-2048-points filter, 5 components over the lipid resonance at 1.5 ppm, Lorentzian line shapes, soft constraints for frequencies and line widths,* |
| c. Output measure  (e.g. absolute concentration, institutional units, ratio) Processing steps deviating from quoted reference or product | *Concentrations in institutional units and pH values*  *Creatine in mM tissue volume*  *^31^P metabolites mM* |
| d. Quantification references and assumptions, fitting model assumptions | *^31^P MR- Quantification relative to ATP, which was assumed to be constant ( [ATP] equal to 8.2mM )*  *depletion during the exercise and end-exercise PCr depletion relative to post-exercise asymptotic value of mono-exponential fit of recovery*  *^1^H MR- Quantification relative to water, fully relaxed water signal was measured separately* |
| 5. Data Quality |  |
| a. Reported variables  (SNR, Linewidth (with reference peaks)) | *SNR taken from AMARES results, noise from residue* |
| b. Data exclusion criteria | *water linewidth after shim> 70 Hz which affects the Cr 3.9 ppm frequency resonance, bad water suppression, baseline distortion* |
| c. Quality measures of postprocessing Model fitting (e.g. CRLB, goodness of fit, SD of residual) | *SD of residual* |
| d. Sample Spectrum | *Figure 2* |
